# Phenotypic plasticity of a winter-diapause mechanism copes with the effects of summer global warming in an ectothermic predator

**DOI:** 10.1098/rsbl.2023.0481

**Published:** 2024-01-17

**Authors:** Hugo Alejandro Álvarez, Francisca Ruano

**Affiliations:** ^1^ Department of Biogeography and Global Change, CSIC – National Museum of Natural Sciences, Madrid, Comunidad de Madrid, Spain; ^2^ Department of Zoology, University of Granada, Granada, Andalucía, Spain

**Keywords:** phenotipic plasticity, global warming, trade-offs, diapause, extreme temperatures

## Abstract

To adapt to changes in temperature, animals tend to invest more energy in thermal tolerance to enhance survival, which can have simultaneous costs on plastic traits. Would a decrease in genetic variability, due to global warming, affect the ability of populations with existing metabolic regulatory mechanisms to cope with extreme temperatures? To address this question, we conducted a series of experiments based on the A1B scenario of global warming, assessing within-population genetic variance in (a) morphological traits, (b) metabolic rate allometries, and (c) survival of a winter-diapausing predator ectotherm. Our study focused on the lacewing species *Chrysoperla pallida*, using both exogamic and endogamic artificial genetic lines. We discovered that both lines use their winter-diapausing phenotype to adapt to summer extreme temperatures caused by extreme heating conditions, but the exogamic line is prone to express phenotypic plasticity in metabolic scaling, with a trade-off between body size and mandible size, i.e. larger individuals tended to develop smaller mandibles to better survive. These findings highlight the significance of substantial phenotypic plasticity and pre-existing metabolic regulatory mechanisms in enabling ectotherms to cope with potential extreme heating occurring in global warming.

## 1. Introduction

Metabolism encompasses the conversion of materials and energy into various biological structures and functions [1,2]. The allometric relationship between metabolic rate and body mass holds significant importance. Allometry refers to the scaling relationship between a specific trait and the size or mass of an organism's body [3–6]. It has been asserted that the allometric slope of the relationship between metabolic rate and body mass remains constant in accordance with the ‘3/4-power law’ [1]. However, in organisms with high activity levels and metabolic constraints imposed by available surface area, the slope tends to exhibit a tendency close to two-thirds, aligning with the surface-to-volume ratio [7]. Furthermore, recent proposals suggest that within natural temperature ranges, metabolic rate may exponentially increase with temperature for a given body size, indicating temperature as a significant source of variation in the allometric slope [8,9].

While the former has been tested on few animal models (e.g. the aquatic crustacean *Daphnia magna* [9]) no attention has been paid to organisms that have already developed mechanisms to cope with severe changes in temperature such as winter diapause—a predetermined condition of developmental arrest, typically accompanied by decreased metabolic activity and enhanced ability to withstand stress [10–12]. For example, it is known that some insects can suppress their metabolic rate thereby allowing them to reduce their reliance on stored food energy, commonly in winter [13]. It is also known that high temperature sensitivity increases in species at higher trophic levels [14], so predators could undergo higher pressures than herbivores when subjected to long periods of extreme temperatures [15].

**Figure 1.**
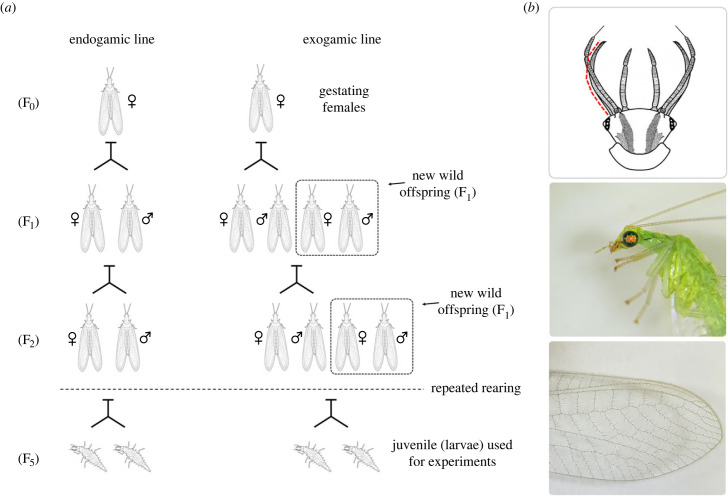
*Chrysoperla pallida*. (*a*) Transgenerational scheme of the genetic lines. (*b*) Larval and adult features; the red line indicates the segmented measurement of mandible length.

In this study, we assessed within-population genetic variance in (a) morphological traits, (b) metabolic rate allometries, and (c) survival, in the larvae of the Meridional-Mediterranean *Chrysoperla pallida* [16–19], a biological-control agent [20] (see details on species biology in electronic supplementary material, S1). To estimate the effects of temperature rising during ontogeny on a transgenerational thermal acclimated population, we designed two genetic lines (i.e. endogamic: inbreed population, and exogamic: outcrossed population of unrelated individuals; figure 1*a*) by using one single female to start the rearing process. This allows the occurrence of (i) low genetic variability whether it was generated in the first female or thanks to the inbreeding in the endogamic line, and (ii) higher genetic variability by recombination with new wild individuals throughout the exogamic line. Then, we performed experiments at different temperatures at the complete developmental stage, imitating the summer natural conditions based on the A1B scenario of global warming, which stablishes a 1.8°C temperature increase by 2050. We tested (i) whether metabolic rates are affected by continuous extreme temperatures during development and (ii) whether there are costs to deal with extreme temperatures by using functional traits such as body mass, mandible length, and survivorship.

## 2. Methods

### (a) Experimental design

Newly hatched larvae from a breeding-stock colony were used for experiments (the offspring of a fifth generation reared at 25° ± 1 to remove maternal effects) (electronic supplementary material, S1). Temperature maxima were established based on monthly mean maxima of summer months: 2016–2018 (sample area). The resulting mean temperature was 37°C, and thus the A1B scenario was set from this temperature, i.e. 38.8°C. We established a control of 26°C—a rounding up based on standard protocols for rearing *Chrysoperla* spp. in the laboratory [21]. These temperatures are in accordance with, and above, natural conditions experienced by *C. pallida* in Mediterranean areas (e.g. from 2003 to 2022: 25°C annual mean maxima versus 35°C mean maxima of hottest month; and 16°C mean annual temperature) (https://www.tutiempo.net/clima/ws-84190.html). Experiments were carried out in an incubator (ICP 600 Memmert). We used the programming function to imitate daily conditions of summer using a 24 h cycle of four consecutive temperature loop stages (electronic supplementary material S1). Individualized larvae inside Petri dishes were reared in the ICP incubator similar to the breeding-stock colony using the same *Ephestia kuehniella* eggs as food, but only providing 6.5 mg of food each time to standardize food availability (electronic supplementary material, S1). Larvae were reared in experimental conditions and hatched within a minimum period of 6 days and a maximum period of approximately 17 days.

Standard metabolic rate (hereafter metabolic rate) was calculated using flow-through respirometry by measuring the rate of carbon dioxide (CO_2_) production. Using the Ideal Gas Law, we converted raw measures (ppm) to molar rates of CO_2_ production (*Ṁ*CO_2_) (electronic supplementary material, S1).

Immediately after metabolic rate measurements were done, the larvae were weighted and the mandible length (left mandible) was measured from photographs when the larvae were relaxed (figure 1*b*). Larvae survival was recorded as the successful pass of a larva to the cocoon stage (pupa).

### (b) Data analysis

To estimate the overall effect of warming temperatures of the A1B scenario on *C. pallida* larvae, firstly, we fitted linear mixed models (LMMs) assessing the effect of the type of genetic line, for which we included the metabolic rate (MR; *Ṁ*CO_2_ of each experimental temperature), body mass (BM; mg) and mandible length (ML; mm) as response variables. For each LMM, the temperature (T) and the genetic line were predictor variables, and the days of experimental rearing were a random effect. Further differences were inspected by a *post-hoc* contrast test on significant model differences of genetic line effects.

Variance in the allometric slopes and intercepts between MR and BM, and between ML and BM, were estimated fitting linear models (LMs) for analysis of covariance (ANCOVA) with log_e_-transformed data to compare OLS regression slopes. We fitted two models (one per genetic line) including as the response variables MR and ML, respectively, and as a predictor variable the interaction BM × T.

Finally, to estimate the effects of different warming temperatures on larvae survival and its relationship with MR, BM, and ML in both lines, we assessed which was the most important variable that could explain larvae survival using a multi-model inference (ΔAIC < 2 [22]). So, we fitted a generalized linear mixed model (GLMM) as a global model, including survival (binary; 1 = alive, 0 = death) as the response variable. The MR, BM, ML, T, and the genetic line were included as predictor variables. The days of experimental rearing were a random effect. Next, each combination of variables in the three experimental temperatures was subjected to a logistic regression approach in order to assess the probability of survival. The resulting significant logistic regressions were used to assess the effects of the interactions BM × MR and BM × ML on survival. All analyses were computed in R software v. 4.0.3 [23] (see details in electronic supplementary material, S1).

## 3. Results

### (a) Overall effects of experimental temperatures

**Figure 2.**
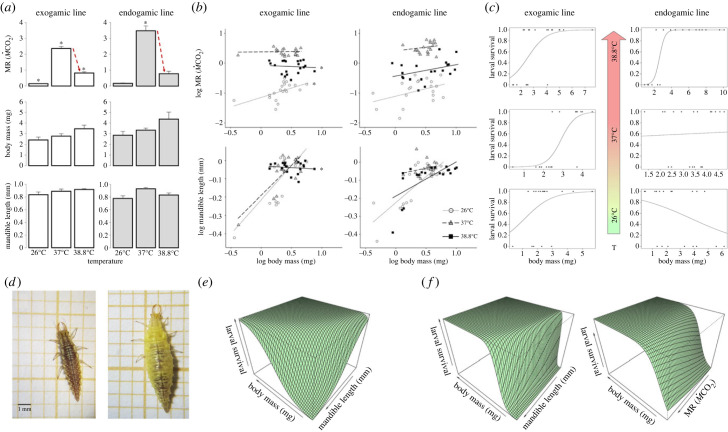
Effects of increasing developmental temperature on *Chrysoperla pallida* larvae. (*a*) Standard metabolic rate (MR), body mass, and mandible length. Bars and error bars represent the mean and standard error, respectively. Red lines indicate the reduction in MR, and thus, the significant difference between genetic lines; asterisks indicate *post-hoc* significant differences. (*b*) Allometric relationship of MR and mandible with body mass, asterisks indicate *post-hoc* significant differences. (*c*) Probability of survival based on the relationship with body mass. (*d*) Third instar larvae at 37°C (left) and 38.8°C (right, displaying the winter–diapause phenotype). Interaction among survival, body mass, and functional traits in both the (*e*) exogamic and (*f*) endogamic lines.

Larvae of both genetic lines changed their behaviour as temperature increased, i.e. larvae at 37°C moved faster inside Petri dishes than the larvae at 26°C, however, at 38.8°C larvae stop moving and changed their colour from brown (figure 2*d*, left) to a strong yellow (figure 2*d*, right). This reflects the species thermal safety margins and shows the typical winter–diapause colour and behavioural response [19].

### (b) Differences among variables

The effect of experimental temperatures was significative for MR, BM, and ML, however, the effect of the genetic line was only significant for the MR (electronic supplementary material, table S1). In both genetic lines, MR increased sharply from 26°C to 37°C and decreased from 37°C to 38.8°C (*post-hoc* contrast test: *α* = 0.05, figure 2*a*). However, the 37°C treatment had a greater effect on MR in the endogamic line compared with the exogamic line (*post-hoc* contrast test: *α* = 0.05, see red lines in figure 2*a*).

### (c) Allometric relationships

The allometric slope of MR and ML was different among experimental temperatures in the exogamic line, i.e. the slopes are not homogeneous among the temperature treatments in these two cases, conversely, the endogamic line showed no difference in the allometric slope of MR and ML, being all homogeneous (electronic supplementary material, table S2). The significant interaction term in the exogamic line was driven by the difference between 26°C and 38.8°C for MR (the slope at 37°C was highly similar to that of 38.8°C), and for ML it was the difference of 38.8°C with both 26°C and 37°C (*post-hoc* contrast test, *α* = 0.05, figure 2*b*). The pattern in both genetic lines did not follow a 3/4 power law tendency (electronic supplementary material, table S2).

### (d) Larval survival

The multi-model inference listed the importance of the variables as: (i) BM, (ii) MR, (iii) T, (iv) ML, and (v) genetic line, being BM and T significative (the former positive and the later negative but marginal; electronic supplementary material, table S3). Thus, we inspected the pattern followed by the relationship between survival and BM across temperatures in logistic regressions (figure 2*c*). For the exogamic line, individuals with larger sizes were more likely to survive at all temperatures, but the number of death-individuals was larger at 38.8°C (small individuals). For the endogamic line, there was a weaker effect in the form of a negative relationship at 26°C, so individuals with larger sizes were less likely to survive. Conversely, at 38.8°C there was an effect in the opposite direction.

The effects of the interactions BM × MR (endogamic line) and BM × ML (both lines) over larval survival were inspected at 38.8°C (due to significant results of logistic regressions, electronic supplementary material, figure S3). The results showed that larger individuals with small mandibles survived more than small individuals with large mandibles in both lines (exogamic: figure 2*e*), however, for the endogamic line there was a strongest effect on BM and a weaker effect on ML (endogamic: figure 2*f*, left). Furthermore, for the endogamic line only, larger individuals tend to survive more at lower metabolic rates (figure 2*f*, right).

## 4. Discussion

We hypothesized that *C. pallida* larvae might use their diapause mechanism to cope with extreme warmer events. For example, we have found that both lines reduced metabolic rate at the highest temperature, so a reduction in metabolism could be a response to extreme high temperatures [24–29], and an adaptive strategy for lacewings. Underlying physiological processes may explain these results under the metabolic theory of ecology [1], e.g. through enzyme inactivation of juvenile hormone [10,30] or through the suppression of metabolism facilitated by a decrease in the production of adenosine triphosphate (ATP) in the mitochondria [12,31]. Recently, it has been discovered that the reduction in the metabolism observed in potato beetles (a diapausing organism) is mediated not by reduced ATP but by mitophagy (mitochondrion-specific autophagy [13]). The mitophagy-induced metabolic suppression showed a decrease in MR highly similar to our results [12].

The exogamic line was prone to show phenotypic plasticity in the metabolic slope. This was reflected in the allometric slopes across temperatures. The pattern can be explained by the ‘acclimation hypothesis for temperature dependence’ [9,32]. According to this new hypothesis, organisms make an effort to mitigate the direct impacts of temperature that may lead to suboptimal reductions or increases in their metabolic rates [33]. However, if there are no acclimation processes, then across different temperatures the allometric slopes should remain parallel suggesting that the thermal plasticity of the slope—and thus the slope, may have limited potential to evolve under these short time-frame-specific circumstances [34]. In this scenario, the plasticity of the regulation in *C. pallida* may be facilitated by the existence of their diapausing mechanism and mediated by genetic variance.

Our results show an extreme scenario, because lacewing larvae may naturally exploit behavioural thermoregulation across the complex mosaic of microclimates produced by living and non-living objects in the environment to avoid thermal extremes [15,35–38], and may never reach the proposed temperatures. This possibility has been hindered by the type of encloser in which individual larvae were reared here, and thus, larvae were pushed to exploit physiological thermoregulation. Indeed, what our study shows is that in the case of no behavioural thermoregulation and individuals facing extreme continuous temperatures (at the hottest hours) throughout the complete developmental stage, pre-existing physiological thermoregulatory mechanisms should cope for such pressures based on phenotypic plasticity and genetic variability. Therefore, by comparing populations with different levels of genetic variation, and to the extent that plasticity is adaptive, one might assume that lack of genetic variation leading to decreased plasticity may be a process of maladaptation.

In this way, ML expressed negative allometry (*b* < 1) at 38.8°C in the exogamic line. As this trait is needed for feeding, allometric theory predicts that it should not express any form of exaggeration [3,39]. A change from isometry to negative allometry in a morphological functional trait would suggest the possibility of a constraint produced by metabolism, as the slope of ML at 38.8°C follows the pattern showed by MR—a link similarly showed by traits functionally associated with the MR [40,41].

In addition, rising temperatures of 1.8°C surpassing summer maxima negatively affected larval survival in our experimental conditions. It is known that survival exhibits the broadest range of sensitivity to temperature [42], but extreme temperatures encountered during the egg, larval, or pupal stages can have a more significant impact on survival than that experienced by adults [43]. We found that BM is the main factor driving the survival of *C. pallida* larvae, i.e. the biggest larvae had the highest probabilities to survive. However, the endogamic line proved to be more sensitive to changes in temperature at higher BM. Enhanced metabolism and fast development commonly produces small individuals via selection for smaller body sizes [44–46]. Nonetheless, the evolutionary benefit of smaller sizes may become a disadvantage when organisms are repeatedly exposed to extreme temperatures, which in turn can lead to the development of larger individuals [47–49] and the expression of trade-offs.

To conclude, the occurrence of phenotypic plasticity in metabolic scaling may not align with the assumption of an independent influence of body size and temperature on MR. In addition, the existence of trade-offs produced by phenotypic plasticity in order to improve survival showed the pressures that some ectotherms may face under extreme climatic heating. Given that pre-existing mechanisms of physiological thermoregulation may cope for the effects of global warming, or at least for thermal extreme events, it is possible that the patterns showed here could be found for other diapausing ectotherms—or even endotherms.

## Data Availability

Data are accessible via Figshare: https://doi.org/10.6084/m9.figshare.23284514 [50]. Supplementary material is available online [51].
